# Improved method for protein complex detection using bottleneck proteins

**DOI:** 10.1186/1472-6947-13-S1-S5

**Published:** 2013-04-05

**Authors:** Jaegyoon Ahn, Dae Hyun Lee, Youngmi Yoon, Yunku Yeu, Sanghyun Park

**Affiliations:** 1 Department of Computer Science, Yonsei University, 3rd Engineering Bldg. 533-1, Shinchon-dong, Seodaemun-gu, Seoul, Korea; 2 Department of Computer Engineering, Gachon University, 1342 Seongnamdaero, Sujeong-gu, Seongnam-si, Gyeonggi-do, Korea

## Abstract

**Background:**

Detecting protein complexes is one of essential and fundamental tasks in understanding various biological functions or processes. Therefore accurate identification of protein complexes is indispensable.

**Methods:**

For more accurate detection of protein complexes, we propose an algorithm which detects dense protein sub-networks of which proteins share closely located bottleneck proteins. The proposed algorithm is capable of finding protein complexes which allow overlapping with each other.

**Results:**

We applied our algorithm to several PPI (Protein-Protein Interaction) networks of Saccharomyces cerevisiae and Homo sapiens, and validated our results using public databases of protein complexes. The prediction accuracy was even more improved over our previous work which used also bottleneck information of the PPI network, but showed limitation when predicting small-sized protein complex detection.

**Conclusions:**

Our algorithm resulted in overlapping protein complexes with significantly improved F1 score over existing algorithms. This result comes from high recall due to effective network search, as well as high precision due to proper use of bottleneck information during the network search.

## Background

Most proteins are known to be involved in complex biological processes or functions in a cell, forming a protein complex with other proteins [1]. Therefore, detecting protein complexes is one of essential and fundamental tasks in understanding various biological functions or processes. A protein complex can be modelled as an undirected graph of which node is a protein and edge is a physical interaction between two protein nodes. This physical interaction of two proteins is called PPI (Protein-Protein Interaction). Representative methods to find those interactions are two-hybrid system [2] and Mass Spectrometry [3]. Recent development of those high-throughput methods has resulted in abundant PPI network.

A protein complex is a set of proteins that interact with each other, so it is frequently assumed that distances between its member proteins are short, and its members tend to form clique-like structure in the PPI network. Accordingly, a protein complex is often assumed as a dense sub-graph in the PPI network. There have been active researches to develop algorithms for detecting protein complexes, and many of them are based on searching dense sub-graph in the PPI network. MCODE [4] gives high weight to nodes of which degree is high, and searches the network using those nodes as seeds. It enforces local search on the network, and finds sub-network whose nodes are highly interconnected. CMC [5] gives weight to PPIs using an iterative scoring method to assess the reliability of PPI, finds maximal cliques from the weighted PPI network, and then removes or merges overlapping maximal cliques based on their interconnectivity. MCL [6] detects clusters by distinguishing the strong and weak connections in the network and partitioning the network, based on manipulation of transition probabilities or stochastic flows between vertices of the graph. MCL has been reported to have good performance, and many variations of it have been proposed [7-9]. However, they are known to suffer from imbalance of resulting clusters [9].

These network clustering algorithms commonly do not allow overlapping between identified protein complexes. In other words, a protein can be involved in only one protein complex. Recently, algorithms that allow overlapping have been extensively studied. DPClus [10] detects initial protein complexes starting from the seeds and then including neighbours so as to maintain the edge's density of the sub-network above the threshold. Then it finds overlapped protein complexes extending the initial protein complexes. CFinder [11] is based on Clique Percolation Method (CPM) [12], which defines a protein complex as a union of k-cliques that share (k-1) vertices. The result of CFinder is sensitive to the value of k. As k increases, it tends to find smaller, but highly denser sub-network. Link Cluster [13] firstly substitutes edges to virtual nodes, and then make edge between those virtual nodes (edges) that share nodes. Virtual nodes of the substituted network are closer as their connectivity increase. Hierarchical clustering of those virtual nodes results in the clusters of the edges, and as a result, those clusters can share nodes. Allowing the overlaps between resulting protein complexes obviously leads to higher recall and precision, because a protein is frequently involved in several protein complexes [10]. Becker et al. [14] proposed Overlapping Cluster Generator (OCG) which decomposes a network into overlapping clusters for correct assignment of multifunctional proteins. The OCG makes initial overlapping classes that are iteratively fused into a hierarchy according to an extension of Newman's modularity function.

Precise prediction of protein complexes is important since they are likely to be fundamental units for various biological functions or processes. Also, the validation cost of predicted protein complexes is high. For more precise detection of protein complexes, we used the characteristics of bottlenecks in the network. A bottleneck of a network is a node that the information of the network is concentrated. The bottleneckness of a node can be calculated using betweenness centrality, which is a measure of a node's centrality in a network, and equal to the number of shortest paths going through it. Yu et al. [15] revealed that bottleneck proteins tend to be essential proteins and correspond to the dynamic component of the PPI network. Moreover, they can be global connectors between functional modules of the PPI network. Therefore, sub-graphs of which boundary proteins are bottleneck proteins have higher chance to be functional modules. We expected that finding these sub-graphs as candidate protein complexes will efficiently filter the possible false predictions out.

Previously, we proposed the protein complex prediction algorithm that utilizes the bottleneck proteins as partitioning points for detecting the protein complexes, based on this expectation [16]. It iteratively constructs directed acyclic graphs of which starting node is bottlenecks in the PPI network. The search ends at nodes where flows from the starting node are concentrated. This graph is called DG (Distance Graph), and terminal nodes of DG tend to be bottlenecks of the PPI network. Established DGs are used to identify sub-graphs that may be overlapped with each other. The sub-graphs having enough edge-density are reported as protein complexes.

Even though [16] showed improved F1 score over previous works, it showed limited results when predicting small-sized protein complexes. For address this problem, we propose new network search algorithm which searches dense protein sub-networks of which proteins share closely located bottleneck proteins.

We applied our algorithm to several PPI networks of Saccharomyces cerevisiae and Homo sapiens, and validated our results using public databases of protein complexes. Our algorithm resulted in significantly improved F1 score over existing algorithms including our previous work [16]. This result comes from high recall due to effective network search, as well as high precision due to proper use of bottleneck information during the network search.

## Methods

The protein complex detection method proposed in this study is composed of two parts. First, betweenness centralities of all the nodes and shortest distances between all node pairs in the PPI network are calculated. Second, we search dense protein sub-networks of which proteins share closely located bottleneck proteins.

The network search starts from sorting nodes by their betweenness centrality in descending order, and putting them in the starting node set. Among them, upper *BC *threshold (user parameter, %) nodes are called bottleneck nodes. Also, each node keeps "close bottlenecks", which is a set of bottleneck nodes of which distance from the nodes ≤ 2.

Each node in the starting node set forms an initial cluster. The initial cluster grows by including neighbouring proteins iteratively, until no nodes can be included. Each cluster keeps its set of shared bottlenecks. In case of the initial cluster, this set means close bottlenecks of its starting node. From each initial cluster, we include neighbouring protein nodes that satisfy two conditions: the edge density and ratio of sharing bottleneck nodes. Given node *n*, these two conditions can be expressed by following score function:

$$\begin{array}{cc} score\left(n\right)= & clutering\;coefficient\;\text{when}\;n\;\text{is}\;\text{included}\;\text{in}\;\text{the}\;\text{cluster}\\ & \begin{array}{c} \times \frac{\text{n}\left(shared\text{\_}bottlenecks\right)}{\text{n}\left(\text{shared}\;\text{bottlenecks}\;\text{of}\;\text{the}\;\text{cluster}\right)}\\ \times \frac{\text{n}\left(shared\text{\_}bottlenecks\right)}{\text{n}\left(\text{colose}\;\text{bottlenecks}\;\text{of}\;n\right)} \end{array} \end{array}$$

"*shared_bottleneck*" indicates intersection of shared bottlenecks of cluster and close bottlenecks of *n*. Edge density can be measured by clustering coefficient, as in our previous work [16].

We find neighbouring nodes from non-bottleneck proteins in the cluster, except for the initial cluster. In other words, bottlenecks are nodes where the search ends. For each neighbouring node that makes clustering coefficient ≥ *CC *threshold, we calculate its score, and include top *k*% scored nodes into next cluster. Throughout the rest of the paper, we used *k *= 5. We used priority queue to implement this mechanism. Using top *k*% scored nodes rather than only one node with best score is essential for efficient network traverse. Higher *k *enables faster clustering, and we confirmed that higher *k *(~ 10%) does not lower the prediction accuracy through iterative experiments.

**Figure 1:**
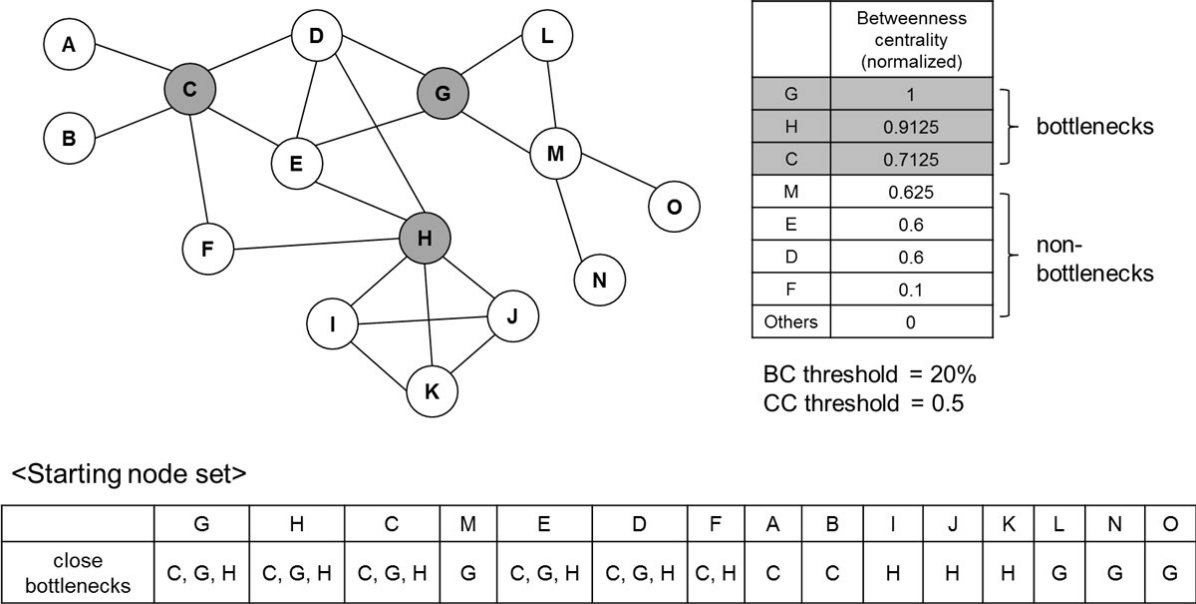
**Example PPI network and bottleneck information**. First, Betweenness centrality of each node in the PPI network is calculated. Protein nodes are sorted according to the betweenness centrality in descending order, and put into starting node set. All nodes keep close bottlenecks, which means distance between node and bottleneck ≤ 2.

**Figure 2:**
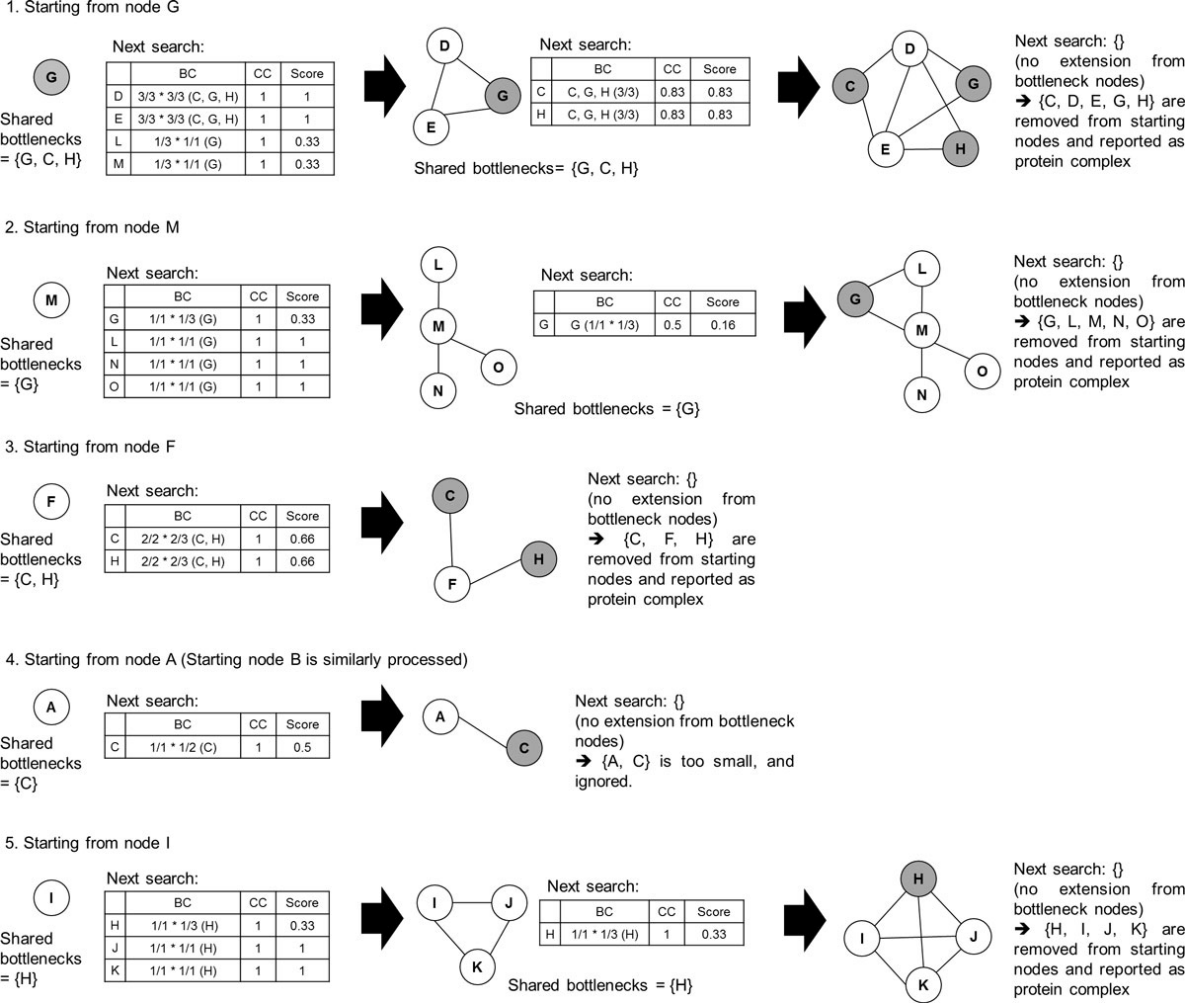
**Detecting protein complexes**. Network searching process for each node of the starting node set in Figure 1. "BC" in the tables indicates second and third term of the score function in the Method chapter. "CC" in the tables indicates clustering coefficient of the cluster when the node is included in the cluster.

Figure 1 shows the example PPI network and its bottleneck nodes. Each node keeps its close bottlenecks. Figure 2 describes search process for the example PPI network. Starting from node G, we can see that its neighbour nodes are D, E, L and M. We calculate the score of them. Cluster {G} has shared bottlenecks {G, C, H}. Node D and cluster {G} share {G, C, H}. So, second term of above formula is 3/3. Node D has close bottlenecks {G, C, H}. So, third term of above formula is 3/3. Because clustering coefficient of {D, G} is 1, *score*(D) is 1. For convenience, we include just top scored nodes, rather than top *k*% scored nodes, into next protein complex in Figure 2. So, initial cluster {G} grows up to {D, E, G}. The neighbouring nodes of those nodes are {C, H}. Because nodes C and H satisfy *CC *threshold, they are included in the cluster. Also, as they are bottlenecks, no neighbouring nodes exist, and the search ends.

**Figure 3:**
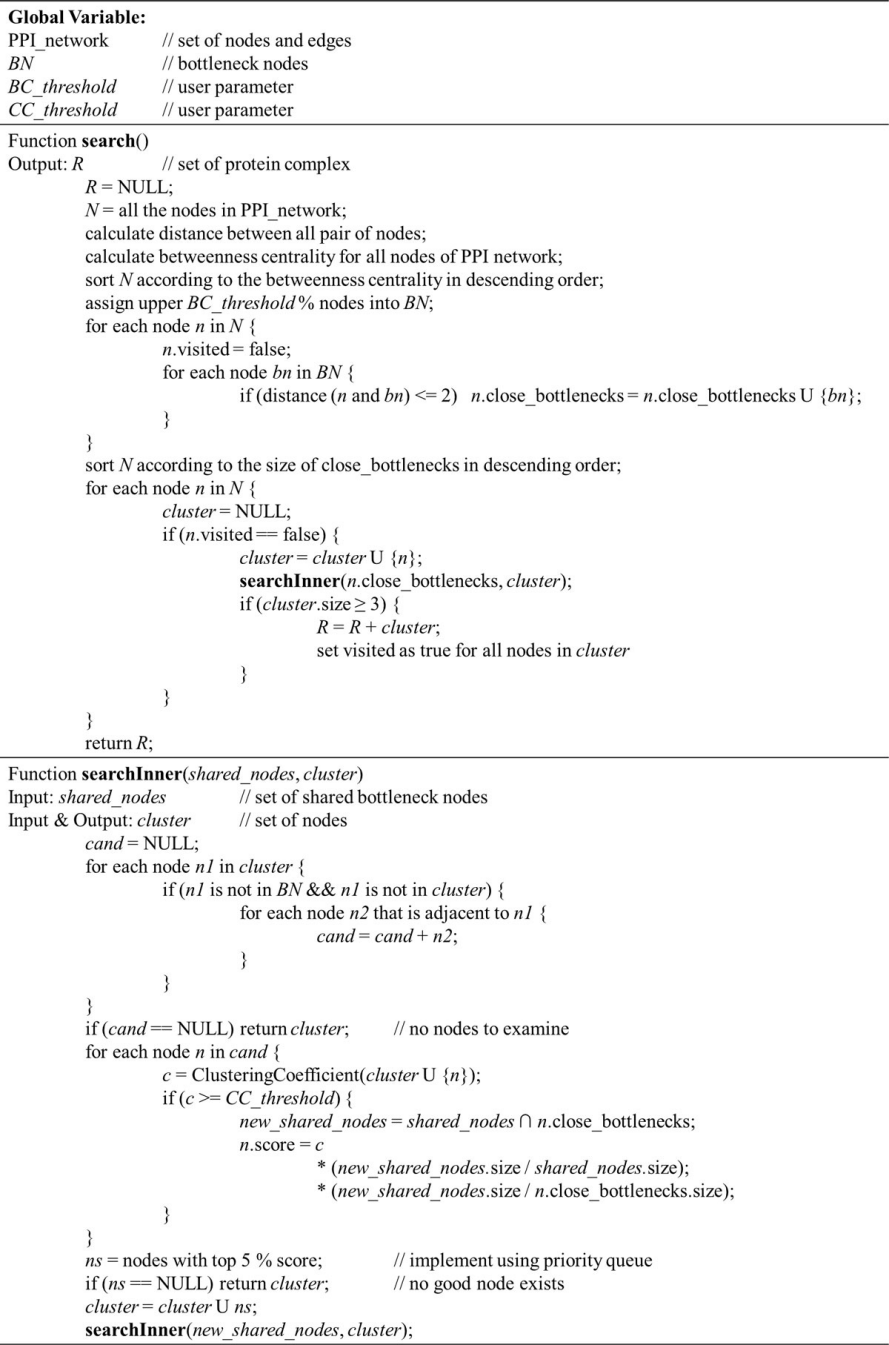
**The pseudo code of the proposed algorithm**.

After searching for the cluster ends, it is reported as protein complex if its size ≥ 3, and its member nodes are removed from the starting node set. This prevents too much overlapping between resulting protein complexes. Figure 3 presents the pseudo code of the described algorithm.

## Results

### Experimental environment

We downloaded two PPI networks of Saccharomyces cerevisiae (yeast) from DIP [17] and BioGRID [18] database. Also, 109,086 human PPIs were downloaded from the I2D database [21]. PPIs from DIP are biologically validated, thus the number of PPIs is relatively small, but they tend to be more accurate. Meanwhile, BioGRID has about ten times more PPIs than DIP. BioGRID has many predicted PPIs, which result in much higher false positive error rate. Table 1 shows the information of the PPI network datasets.

**Table 1:** PPI network datasets

Database (version)	Species	Number of proteins	Number of PPIs
DIP (20071007)	Saccharomyces cerevisiae	4,823	16,914
BioGRID (3.1.69)	Saccharomyces cerevisiae	5,920	162,378
I2D (1.95)	Homo Sapiens	14,610	209,440

We also collected known protein complexes (reference) to validate the results of our algorithm. Two reference datasets of Saccharomyces cerevisiae were downloaded from MIPS [19] and CYC2008 [20] database. One reference dataset of Homo sapiens was downloaded from CORUM database [22]. For both reference datasets and identified protein complex sets, we used complexes of which size is more than or equal to three. Table 2 shows the information of collected reference datasets.

**Table 2:** Reference datasets

Database (version)	Species	Number of protein complexes	Number of proteins	Avg. number of proteins in protein complexes
MIPS	Saccharomyces cerevisiae	81	885	12.358
CYC2008 (2.0)	Saccharomyces cerevisiae	236	1,627	6.678
CORUM (17.02.2012)	Homo Sapiens	1,942	4,394	5.789

### Performance test

To see whether a complex identified by an algorithm is matched with protein complexes in the reference datasets, we used affinity score. Given set of proteins in a protein complex in a reference dataset and set of proteins in an identified protein complex, which we call A and B respectively, affinity score between A and B can be calculated by the following Equation.

$$aff\left(\text{A},\;\text{B}\right)=\text{n}{\left(\text{A}\cap \text{B}\right)}^{2}/\left(\text{n}\left(\text{A}\right)\times \text{n}\left(\text{B}\right)\right)$$

The searching is successful if a protein complex is identified with affinity score ≥ 0.2 for any protein complex in a reference datasets. If this threshold is too big or small, the affinity score loses its assessment function. Through iterative experiments, we set the affinity score threshold as 0.2, which makes the difference between results of various algorithms.

The performance of a clustering algorithm can be measured using recall, precision and F1 score, which are calculated as follows:

$$\begin{array}{lll} \text{Recall}=\left|R_{hit}\right|/\left|\text{R}\right|,\text{Precision}=\left|C_{hit}\right|/\left|\text{C}\right|, & \; & \\ \text{F}1\;\text{score}=\text{harmonic}\;\text{mean}\;\text{of}\;\text{Recall}\;\text{and}\;\text{Precision}, & \; & \\ R_{hit}=\left\{R_{i}\in R|aff\left(R_{i},C_{j}\right)\geq 0.2,C_{j}\in C\right\}, & \; & \\ C_{hit}=\left\{C_{i}\in C|aff\left(C_{i},R_{j}\right)\geq 0.2,R_{j}\in R\right\}, & \; & \\ & \; & \end{array}$$

where *C *is a set of protein complexes found by a clustering algorithm, and *R *is a set of protein complexes in a reference dataset. Recall means a rate of protein complexes in the reference datasets that were successfully found, precision means a rate of protein complexes identified by an algorithm that are matched with the protein complexes in the reference datasets, and F1 score means an overall accuracy of the test.

**Figure 4:**
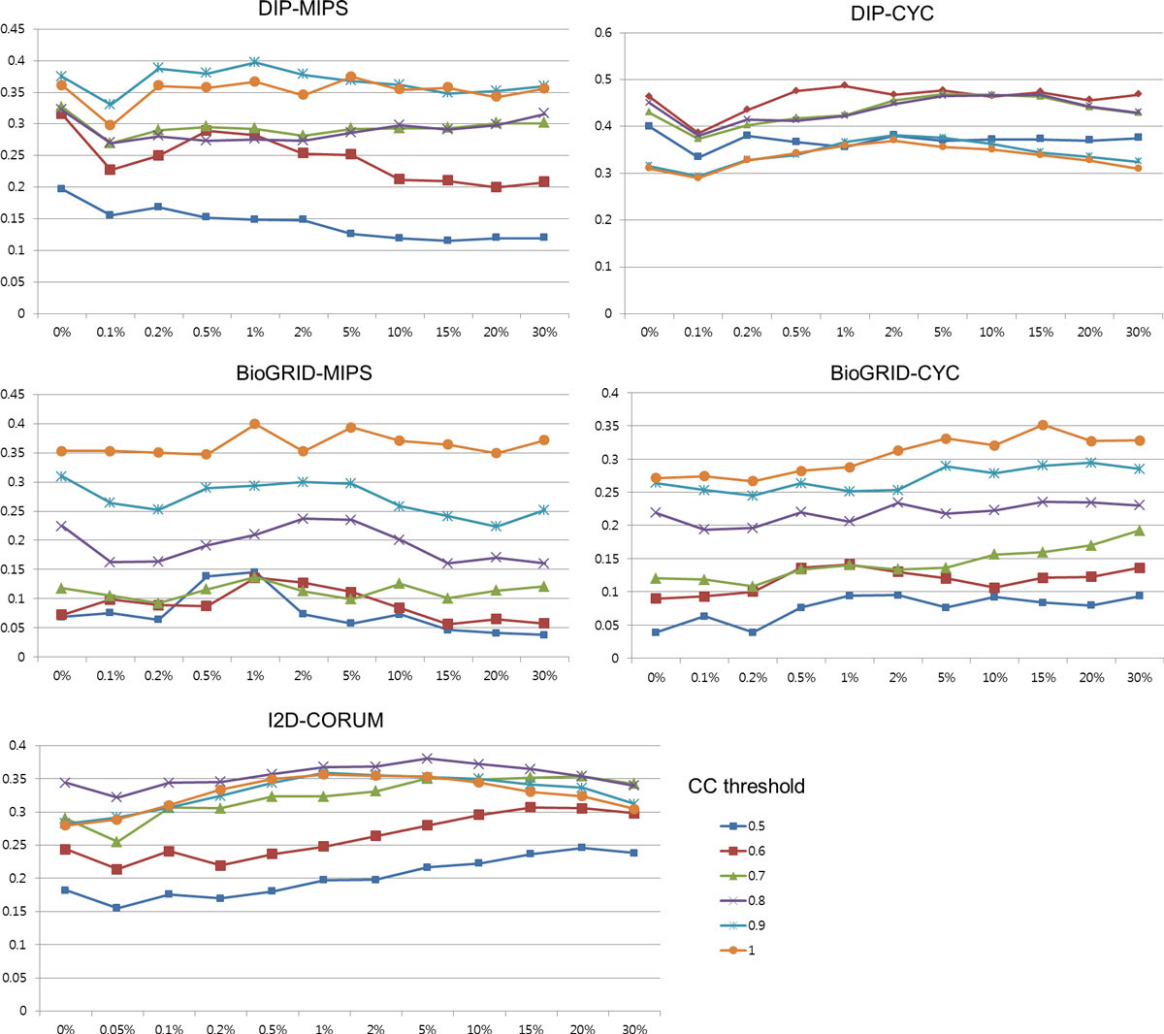
**Experimental results for obtaining optimal user parameters**. Each title of the graph indicates "PPI network dataset - reference dataset". X and Y axis indicate *BC *threshold and F1 score, respectively. Zero *BC *threshold means that we did not use any bottleneck proteins.

First, we tested the performance of proposed algorithm varying two user parameters, *BC *and *CC*. The results are shown in Figure 4. The optimal *CC *and *BC *thresholds are from 0.6 to 0.8 and from 1%~5% respectively, for three experiments using DIP and I2D datasets (DIP-MIPS, DIP-CYC and I2D-CORUM). For two experiments using BioGRID dataset, the optimal *CC *and *BC *thresholds are from 1% to 15% and 1.0, respectively. The supposed reason of these differences in optimal thresholds is that BioGRID has large number of predicted PPI, which leads to higher false positive complex predictions. Therefore, the precision would decrease unless *CC *is high enough, as shown in these two graphs. For the same reason, relatively large number of bottleneck seems to be helpful for accurate prediction.

To see the impact of using bottlenecks, we performed experiments using only clustering coefficient, which means *score *function in Methods chapter is as follow:

$$score\left(n\right)=clutering\;coefficient\;\text{when}\;n\;\text{is}\;\text{included}\;\text{in}\;\text{the}\;\text{cluster}$$

For all the experiments, tests using bottleneck information brought more accurate results. Especially, prediction accuracies were clearly increased when using bottlenecks in two cases using BioGRID. This means that bottleneck information were effective in dense network which may include many false interactions. At the same time, tests using only clustering coefficient shows comparable prediction accuracy, which means that the proposed network searching algorithm is effective for detecting protein complexes.

We then measured the prediction performance of proposed algorithm, and compared the results with representative network clustering algorithms, MCODE [4], MCL [5], Link Cluster [13], and our previous work [16]. We applied each algorithm including proposed algorithm to PPI networks and two reference datasets. For each algorithm, we found optimal parameters that result in best F1 score.

In Table 3, the proposed algorithm shows overall high F1 score. Except for DIP-MIPS experiment, F1 score of the proposed algorithm is significantly improved over our previous work [16]. Our previous work showed limited performance on finding small-sized protein complexes, as shown in experiments DIP-CYC, BioGRID-CYC and I2D-CORUM. While high precision was the strength of [16], we can confirm that the increased F1 score comes from higher recall, as well as high precision.

**Table 3:** Result of comparison test

PPI network dataset	Reference dataset	Algorithm	Optimal parameters	Number of protein complexes	Recall	Precision	F1 score
DIP	MIPS	Proposed	*CC *= 0.9, *BC *= 1%	269	0.5556	0.3086	0.3968
		[16]	*CC *= 0.51, *BC *= 20%	76	0.3210	0.4605	0.3783
		Link Cluster	Partition_density = 0.30	1,177	0.7037	0.1427	0.2373
		MCL	Granularity = 2.00	614	0.5679	0.0739	0.1298
		MCODE	Node_score = 0.10	83	0.2930	0.2530	0.2729
	
	CYC	Proposed	*CC *= 0.6, *BC *= 1%	646	0.4877	0.4860	0.4869
		[16]	*CC *= 0.38, *BC *= 20%	333	0.3898	0.4114	0.4003
		Link Cluster	Partition_density = 0.29	1,179	0.5932	0.2858	0.3857
		MCL	Granularity = 2.40	639	0.4746	0.1690	0.2493
		MCODE	Node_score = 0.10	83	0.2119	0.5542	0.3065

Bio-GRID	MIPS	Proposed.	*CC *= 1.0, *BC *= 1%	127	0.3457	0.4724	0.3709
		[16]	*CC *= 0.54, *BC *= 20%	69	0.2346	0.3623	0.2848
		Link Cluster	Partition_density = 0.30	10,463	0.5926	0.0893	0.1552
		MCL	Granularity = 3.60	216	0.2099	0.0556	0.0879
		MCODE	Node_score = 0.10	120	0.086	0.0500	0.0633
	
	CYC	Proposed	*CC *= 1.0, *BC *= 15%	506	0.3260	0.3814	0.3515
		[16]	*CC *= 0.43, *BC *= 30%	324	0.2500	0.2160	0.2318
		Link Cluster	Partition_density = 0.28	10,915	0.5297	0.2802	0.3697
		MCL	Granularity = 3.00	225	0.1144	0.1111	0.1127
		MCODE	Node_score = 0.10	120	0.0593	0.1167	0.0787

I2D	CORUM	Proposed	*CC *= 0.8, *BC *= 5%	2,508	0.4100	0.3545	0.3802
		[16]	*CC *= 0.41, *BC *= 20%	1,132	0.2961	0.2491	0.2706
		Link Cluster	Partition_density = 0.21	8,033	0.4576	0.1595	0.2378
		MCL	Granularity = 1.60	750	0.0623	0.0587	0.0604
		MCODE	Node_score = 0.10	251	0.0469	0.1076	0.0652

We can see that optimal *BC *thresholds are generally smaller, and optimal *CC *thresholds are higher than [16]. This indicates the proposed algorithm detects denser sub-network. However, this does not means that the proposed algorithm uses less bottleneck information, because prediction accuracy was also good for higher *BC*. Because our algorithm uses bottlenecks as boundary of the protein complex, detected sub-networks are basically similar to the DG. However, division procedure of DG [16] has limitation on detecting dense sub-network. Therefore, we can say that the network searching algorithm we proposed overcame the limitation when detecting dense sub-networks.

**Figure 5:**
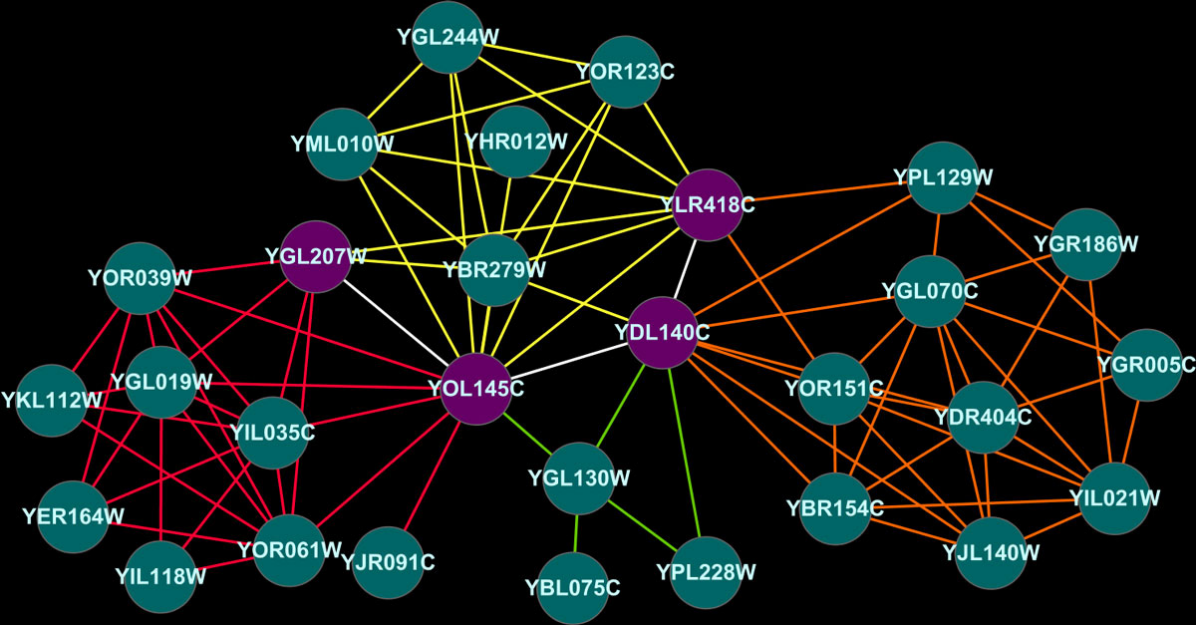
**Example protein complexes**. White interactions indicate shared PPI between protein complexes. Purple nodes are bottleneck nodes. Protein complexes were obtained from DIP dataset and annotated using GO database (p-value < 0.01). Red interactions are core mediator complex, orange interactions are ubiquintin conjugating enzyme complex, yellow interactions are negative cofactor 2 complex and lime interactions are transcription factor TFIIF complex.

Like [16], the proposed algorithm can detect protein complexes that shares PPIs. We can see that overlapped region of different protein complexes contains PPIs in Figure 5. Also, we can confirm that bottleneck proteins function as boundaries for protein complexes.

## Conclusions

We proposed the novel network clustering algorithm which detects dense protein sub-networks of which proteins share closely located bottleneck proteins. The proposed algorithm showed improved F1 score which comes from high recall due to effective network search, as well as high precision due to proper use of bottleneck information during the network search.

As future works, we extend our algorithm to detect the hierarchical relationship between sub-networks identified. This algorithm would help us to elucidate hierarchical structure of various protein complexes or functional modules in a cell, and to infer a function of them in conjunction with various biology databases such as Gene Ontology database.

## Competing interests

The authors declare that they have no competing interests.

## Authors' contributions

JA designed the algorithm, developed application, executed experiments, analyzed the data and wrote the paper. DHL developed application and executed experiments. YYO contributed to design of experiments and wrote the paper. YYE executed experiments, analyzed the data. SP contributed to design of the algorithm and experiments, analyzed the data and wrote the paper.

## Declarations

The publication costs for this article were funded by the corresponding author.

This article has been published as part of *BMC Medical Informatics and Decision Making *Volume 13 Supplement 1, 2013: Proceedings of the ACM Sixth International Workshop on Data and Text Mining in Biomedical Informatics (DTMBio 2012). The full contents of the supplement are available online at http://www.biomedcentral.com/bmcmedinformdecismak/supplements/13/S1.
